# Does exercise improve healing of diabetic foot ulcers? A systematic review

**DOI:** 10.1186/s13047-021-00456-w

**Published:** 2021-03-20

**Authors:** Morica M. Tran, Melanie N. Haley

**Affiliations:** 1 Department of Podiatry, Eastern Health, Melbourne, Victoria 3128 Australia; 2 Department of Aged and Complex Medicine, Eastern Health, Melbourne, Victoria 3128 Australia

**Keywords:** Diabetic foot ulcer, Exercise, Physical therapy, Wound healing

## Abstract

**Background:**

For patients with diabetic foot ulcers, offloading is one crucial aspect of treatment and aims to redistribute pressure away from the ulcer site. In addition to offloading strategies, patients are often advised to reduce their activity levels. Consequently, patients may avoid exercise altogether. However, it has been suggested that exercise induces an increase in vasodilation and tissue blood flow, which may potentially facilitate ulcer healing. The aim of this systematic review was to determine whether exercise improves healing of diabetic foot ulcers.

**Review:**

We conducted a systematic search of MEDLINE, CINAHL and EMBASE between July 6, 2009 and July 6, 2019 using the key terms and subject headings diabetes, diabetic foot, physical activity, exercise, resistance training and wound healing. Randomised controlled trials were included in this review.

Three randomised controlled trials (139 participants) were included in this systematic review. All studies incorporated a form of non-weight bearing exercise as the intervention over a 12-week period. One study conducted the intervention in a supervised setting, while two studies conducted the intervention in an unsupervised setting. Two studies found greater improvement in percentage wound size reduction in the intervention group compared with the control group, with one of these studies achieving statistically significant findings (*p* < 0.05). The results of the third study demonstrated statistically significant findings for total wound size reduction (*p* < 0.05), however results were analysed within each treatment group and not between groups.

**Conclusion:**

This systematic review found there is insufficient evidence to conclusively support non-weight bearing exercise as an intervention to improve healing of diabetic foot ulcers. Regardless, the results demonstrate some degree of wound size reduction and there were no negative consequences of the intervention for the participants. Given the potential benefits of exercise on patient health and wellbeing, non-weight bearing exercise should be encouraged as part of the management plan for treatment of diabetic foot ulcers. Further research is required to better understand the relationship between exercise and healing of diabetic foot ulcers.

**Supplementary Information:**

The online version contains supplementary material available at 10.1186/s13047-021-00456-w.

## Background

Diabetic foot ulcers (DFUs) are a serious and devastating complication of diabetes, affecting 26 million people worldwide annually [1]. People with diabetes have an approximate 25% lifetime risk of developing a foot ulcer compared to those without diabetes [2–4], and prevalence has been reported at 4–10% of the diabetic population [5, 6]. DFUs develop following injury, usually in the presence of peripheral neuropathy, ischaemia or both [2, 7]. The initial ulcer may be precipitated by acute, chronic repetitive or continuously applied mechanical stress, or thermal trauma [7]. Approximately 50% of DFUs occur on the plantar aspect of the foot [8] and if not treated appropriately, can progress into chronic and non-healing ulcers [2]. DFUs are a recognised risk factor for poor health outcomes, including major limb amputation [9–11], and are also associated with a financial burden to the health care system due to extensive healing times [12], reduced quality of life and an increased rate of mortality [13].

Management of DFUs include treatment of foot infection, appropriate dressing plans with regular sharp debridement of nonviable tissue, revascularisation (if indicated), and pressure offloading [14]. Offloading is one crucial aspect of treatment and aims to redistribute pressure away from the ulcer site [1], thereby, reducing further tissue trauma and facilitating the wound healing process [15]. This can be achieved via an offloading device, such as a total contact cast (TCC) or a controlled ankle motion (CAM) walker [1]. In addition to offloading strategies, patients are often advised to reduce their activity levels [16–18]. Consequently, patients may avoid exercise altogether [19]. However, exercise is important for overall heath and may reduce the risks of developing cardiovascular diseases [20]. In relation to the diabetic population specifically, inactivity may lead to diabetes macrovascular and microvascular complications, including ischaemic heart disease, cerebrovascular disease, peripheral vascular diease, retinopathy, nephropathy and peripheral neuropathy [21].

Inactivity is one modifiable risk factor for developing diabetes macrovascular and microvascular complications [21]. The Action in Diabetes and Vascular Disease Preterax and Diamicron MR Controlled Evaluation (ADVANCE) randomised controlled trial (RCT) study [22] reports a strong association between moderate and rigorous physical activity with a reduced incidence of cardiovascular events, microvascular complications, as well as all-cause mortality in participants with type 2 diabetes. However, this RCT did not include participants with DFUs and literature regarding the association between physical activity and vascular complications is limited [23].

The mechanism of exercise on healing of DFUs is not well investigated. In the diabetic population, hyperglycaemia inhibits nitric oxide (NO) synthesis, affecting insulin resistance and reducing the vasodilator response in blood vessels [24]. A meta-analysis by Qiu et al. [25] suggests that exercise induces an increase in blood flow, leading to an increase in NO synthesis and reducing oxidative stress in persons with type 2 diabetes. The combination of vasodilation and increase in tissue blood flow may potentially facilitate ulcer healing [24–27].

The International Working Group on the Diabetic Foot (IWGDF) guidelines [28] support various forms of foot-related exercises, such as strengthening and stretching, to improve modifiable risk factors for incidence of foot ulceration [29–38]. These exercises aim to improve plantar pressure distribution, neuropathy symptoms, reduced foot sensation and foot-ankle joint mobility [29–38]. However, where there are pre-ulcerative lesions or active ulceration, it is recommended weight bearing or foot-related exercises should be avoided [1].

To our knowledge, there is currently no systematic review investigating the effect of exercise and healing of DFUs. A systematic review published by Matos et al. [39] explores physical activity and exercise on diabetic foot related outcomes. However, the outcomes of interest were not specific to wound healing. A second systematic review published by Aagaard et al. [40] investigates the benefits and harms of exercise and DFUs. This study examines exercise and quality of life and adverse events and outcomes of exercise in relation to DFUs. Though this paper does not analyse the effects of exercise and wound healing, it highlights the need for further well-conducted RCTs to guide rehabilitation, including exercise in a semi-supervised and supervised setting [40].

The purpose of this review was to systematically identify, critique and evaluate literature investigating the effect of exercise and healing of DFUs. The primary outcome measure was wound size reduction. The secondary outcome measures were adherence to exercise, complications and adverse events.

## Method

### Registration

This systematic review was prospectively registered on PROSPERO (Registration No. 147487).

### Eligibility criteria

#### Study design

To ensure the highest quality of evidence, only RCTs were included in this review.

#### Population

The RCTs could be conducted in any setting, including, but not limited to, hospital, private clinics, community settings, or within the participant’s home setting. Participants had to be 18 years and over, diagnosed with either type 1 or type 2 diabetes mellitus, have an active foot ulcer and attending a diabetic foot service.

#### Intervention

The intervention included any form of physical activity that was prescribed and measured by a health professional or member of the research team. This included provision of a prescribed exercise sheet.

#### Comparator

The comparator of interest was usual care.

#### Outcome

The primary outcome of interest was wound size reduction (measured in %, cm^2^ or cm). The secondary outcomes of interest were adherence to exercise, complications and adverse events.

#### Information sources

Electronic database searches were conducted in MEDLINE, CINAHL and EMBASE between July 6, 2009 and July 6, 2019 using the key terms and subject headings diabetes mellitus, diabetes, foot disease, diabetic foot, physical activity, exercise therapy, exercise, resistance training, physical fitness, physical therapy, aerobic exercise, exercise therapy, wound, foot ulcer, pressure ulcer, foot ulcer and wound healing. The searches were performed on the three selected electronic databases as they are commonly used databases, and most relevant to the subject of DFUs. A 10-year time period was applied to the search strategy to ensure currency of literature. The search strategy for MEDLINE is shown in Additional file 1.

The reference lists of included studies were checked and citation tracking was performed using Google Scholar to further identify relevant articles for inclusion. Searches were performed again in December 2020 to ensure any new citations were identified and assessed prior to submission.

#### Study selection

The titles and abstracts of records identified in the search strategy were independently screened by two reviewers (MT and MH) based on the inclusion and exclusion criteria. Full text articles were obtained for articles where a decision could not be made to include or exclude. Any disparities were discussed until consensus was reached.

#### Data extraction

A customised tool was created and utilised to extract data from included studies. Information extracted included author, population, setting, details of randomisation, description of exercise intervention, frequency or intensity of intervention, duration of intervention, delivery mode of intervention, description of control and interventions groups, outcome measurements for control and intervention groups, and results of analysis. The customised tool is shown in Additional file 2.

One reviewer performed data extraction (MT), while a second reviewer (MH) confirmed the extracted data. Any disparities were discussed until consensus was reached.

#### Risk of bias in individual studies

Methodological quality of included studies was assessed using the Physiotherapy Evidence Database (PEDro) Scale. This scale consists of 11 items and has been shown to have fair to good reliability [41]. Item 1 pertains to external validity and is not used to calculate the PEDro score (as outlined in the PEDro guideline). Each criterion is given a score of 1 or 0, with a maximum achievable score of 10. Studies with a score equal to or greater than seven indicates high methodological quality, a score between four and six (inclusive) indicates moderate methodological quality, and a score equal to or below three indicates low methodological quality [41]. Studies are critiqued based on the following characteristics:

- Random allocation to groups;
- Allocation concealment;
- Similar baseline characteristics, regarding the most important prognostic indicator;
- Blinding of subjects, assessors and therapists;
- Measurement of at least one key outcome obtained for more than 85% of the subjects initially allocated to groups;
- All subjects received the treatment or control condition as allocated, or where this was not the case, data for at least one key outcome was analysed by ‘intention to treat’;
- Between group statistical comparisons reported for at least one key outcome;
- Provision of point measures and measures of variability for at least one key outcome [42].

Two reviewers (MT and MH) independently applied the PEDro scale to the included studies. Any discrepancies were discussed until consensus was reached.

#### Summary measures

All studies were analysed descriptively and incorporated mean, median and standard deviation (SD).

## Results

### Study selection

**Fig. 1:**
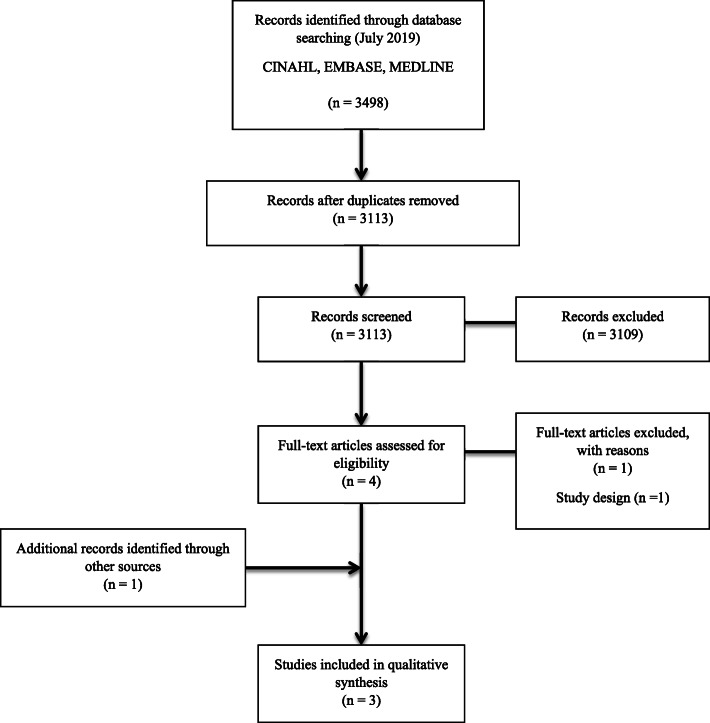
Flow diagram presenting the process undertaken to identify eligible studies

The results of the search process are shown in Fig. 1. The initial search strategy yielded 3498 results across the three databases. Following the removal of duplications and screening of titles and abstracts, four studies were identified for full text review. On review of the full text articles, two studies met the inclusion criteria. Citation tracking was performed on the two included studies, identifying one additional study for inclusion. Therefore, a total of three studies were included in this review.

### Study characteristics

Three RCTs (139 participants), of which one was a pilot study [43], were included in this review. There were 71 participants allocated to the intervention groups and 68 participants allocated to the control groups. The duration for all study interventions was 12 weeks. The mean age of participants in the intervention groups were 61.90 [43], 61.03 [44], and 69.06 [45]. The mean age of participants in the control groups were 74.25 [43], 65.76 [44], and 68.50 [45]. The age range of all participants was 41 to 94 years. Characteristics of included studies are presented in Table 1. A detailed table of the extracted data (including individual study results) is available in Additional file 3.

**Table 1:** Study characteristics of included studies

Author	Participants/Age (Years)			Mean Diabetes Duration (Years)	Presence of Neuropathy	Previous Ulceration	BMI (Kg/m^2^)	Presence of Co-Morbidities
	Intervention Group (IG)	Control Group (CG)	Male					
Flahr [43]	10 Mean age = 61.9 Age range = 49–74	8 Mean age = 74.25 Age range = 54–94	67%	Not reported	50% of participants reported to have 100% loss of sensory^a^	Not reported	Not reported	IG = 6^b^ CG = 3^c^
Eraydin and Avsar [44]	30 Mean age = 61.03 ± 9.97 Age range = 41–80	30 Mean age = 65.76 ± 8.57 Age range = 49–80	62%	IG = 16.23 ± 8.57 CG = 17.46 ± 8.79	Not reported	70%	IG = 31.36 ± 7.62 CG = 28.58 ± 4.66	Not reported
Joseph et al. [45]	31 Mean age = 69.06 ± 4.79 Age range = Not reported	30 Mean age: 68.50 ± 5.01 Age range = Not reported	51%	IG = 21.77 ± 7.77 CG = 18.73 ± 7.16	Not reported	Not reported	IG = 27.66 ± 5.44 CG = 22.96 ± 3.23	Not reported
**Author**	**Setting**	**Description of Intervention**	**Frequency/Duration of Intervention/Delivery Mode of Intervention**	**Outcome Measures**	**Primary Outcome**	**Secondary Outcome**		
					**Wound Measurements** ***IG* = Intervention Group** ***CG* = Control Group**	**Adherence to Exercise in Intervention Group/Complication and Adverse Events**		
Flahr [43]	Home	Non-weight bearing exercises including ankle inversion, eversion, flexion and extension - 4 in total.	10 times each, twice daily 12 weeks Education session in clinic. Provision of written material. Unsupervised exercise	Percentage wound size reduction by participant, self-reported number of days of exercise frequency	Final wound measurement and percentage wound size reduction after 12 weeks: IG1 = 0.22 cm^2^ (− 88%); CG1 = 0.79 cm^2^ (+ 25%) IG2 = Withdrew (− 59%); CG2 = 0.49 cm^2^ (+ 14%) IG3 = 0.09 cm^2^ (− 67%); CG3 = 0.14 cm^2^ (− 88%) IG4 = Closed (− 100%); CG4 = Closed (− 100%) IG5 = 0.12 cm^2^ (− 25%); CG5 = 9.18 cm^2^ (+ 2%) IG6 = Closed (− 100%); C6G = Closed (− 100%) IG7 = 0.05 cm^2^ (− 69%); CG7 = Withdrew IG8 = 0.09 cm^2^ (− 67%); CG8 = Closed (− 100%) IG9 = Closed (− 100%); CG9 = 0.06 cm^2^ (− 95%) IG10 = 2.36 cm^2^ (− 131%) (*p* = 0.70)	1 time/day = 1 (10.0%) 2 times/day = 2 (20.0%) 3 times/day = 1 (10.0%) 2 times every 3rd day = 1 (10.0%) Stopped after 8 weeks = 2 (20.0%) Didn’t exercise = 1 (10.0%) Unknown = 2 (20.0%) 1 participant in IG withdrew due to Osteomyelitis		
Eraydin and Avsar [44]	Home	Non-weight bearing foot exercises to be completed seated: plantar flexion, dorsiflexion, inversion, eversion, circumduction and plantar dorsiflexion of toes - 18 in total. Exercises to be completed standing once wounds healed.	10 repeats, twice daily 12 weeks 20–30 min education session in clinic. Provision of written material. Unsupervised exercise	Mean DFU area, DFU total depth, self-reported exercise frequency	Baseline and final measurements: Distribution of DFU Area Averages (SD): IG: 12.63 cm^2^ (14.43) IG: 3.29 cm^2^ (3.80) (*p* = 0.00)^d^ CG: 24.67 cm^2^ (20.70) CG: 18.52 cm^2^ (21.49) (*p* = 0.00) ^d^ Distribution of DFU Total Depth (SD): IG: 0.56 cm (0.85) IG: 0.28 cm (0.38) (*p* = 0.01) ^d^ CG: 0.61 cm (0.84) CG: 0.80 cm (1.26) (*p* = 0.37) ^d^	0–30 days = 8 (26.7%) 31–60 days = 15 (50.0%) 61–90 days = 7 (23.3%)		
Joseph et al. [45]	Exercise clinic	Participants rode on a bicycle ergometer with foot interaction kept constant with standard gym pedal and specialised offloading insole padding to relieve pressure to ulcer.	3 times per week at exercise clinic 12 weeks Participants encouraged to increase their exercise time by 5 mins each 2 weeks until they reach 50 mins at the 9th week, which was maintained until the end of the program. Supervised exercise	Percentage wound size reduction	Percentage Wound Size Reduction after 12 weeks (SD): IG: 94.08% (18.50) CG: 54.76% (17.19) (*p* < 0.05)	Not reported		

^a^Data break down not available

^b^ 30% had arthritis, 10% had a history of cerebral vascular incident, 10% had a history of back surgery, 10% reported a history of a herniated disc

^c^ 38.5% had arthritis

^d^Intragroup comparison

All three studies incorporated a form of non-weight bearing exercise as the intervention [43–45]. Two studies [43, 44] investigated the effect of prescribed non-weight bearing exercise in the home setting, while one study [45] investigated the effect of supervised non-weight bearing aerobic exercise at a clinic.

For the two studies that prescribed non-weight bearing exercises in the home setting, exercises were performed ten times, twice daily and consisted of different exercise protocols [43, 44]. The exercises required participants to be in a seated position and included plantar flexion, dorsiflexion, inversion, eversion and circumduction of the foot, and plantar and dorsiflexion of the toes. Both studies required participants to keep an exercise log [43, 44]. In contrast, the supervised non-weight bearing aerobic exercises were performed three times weekly, for up to 50 min per session [45]. In this study, participants attended an exercise clinic where they were required to ride a bicycle ergometer, while using an insole pad to offload the DFU during exercise [45]. All participants in the control groups received usual care.

All three studies measured wound healing [43–45]. Two studies measured percentage wound size reduction [43, 45], and one study measured total wound size reduction (cm^2^) as well as wound depth (cm) [44]. Two studies reported participants’ self-adherence to exercise [43, 44].

As the studies differed in the way they prescribed exercises and measured the outcomes of interest, the results could not be pooled in a meta-analysis.

### Risk of bias within studies

**Fig. 2:**
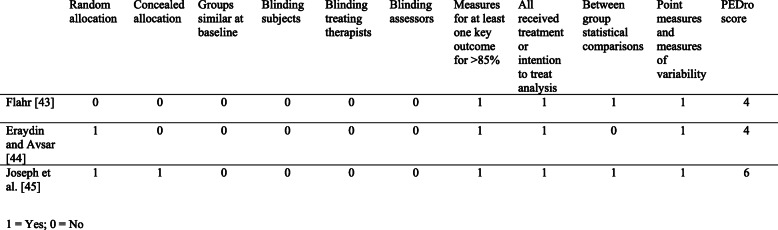
PEDro scale scoring

The quality assessment scores obtained ranged between four and six, indicating moderate methodological quality of included studies. As it is not possible to blind both the participant and treating therapist, studies in this review were not able to achieve a score greater than eight out of ten on the PEDro scale. The PEDro scale scoring of the included studies is shown in Fig. 2.

### Percentage reduction of wound size

Two studies [43, 45] measured percentage reduction of wound size.

One study [43] found no significant difference in percentage wound size reduction between both study arms (*p* = 0.70). In this study, 90% of the intervention group (*n* = 9) experienced a reduction in wound size of 26 to 100% after 12 weeks, compared to the control group where 33% of participants (*n* = 3) experienced 31% increase in wound size.

Another study [45] reported significantly greater percentage reduction in wound size in the intervention group compared to the control groups. In this study, the mean percentage wound size reduction after 12 weeks was 94.08% (SD, 18.50) and 54.76% (SD, 17.19) in the intervention and control groups respectively (*p* < 0.05).

### Total wound size reduction

One study [44] measured total wound size reduction (cm^2^). The results of this study were reported within groups only and did not compare results between both study arms. In this study, both intervention and control groups had significantly reduced wound areas between baseline and 12 weeks (*p* < 0.05). The mean total wound size improved from 12.63cm^2^ (SD, 14.43) to 3.29cm^2^ (SD, 3.80) in the intervention group (*p* = 0.00), compared with an improvement of 24.67cm^2^ (SD, 20.70) to 18.52cm^2^ (SD, 21.49) in the control group (*p* = 0.00) [44].

### Total wound depth

One study [44] measured total wound depth and conducted intragroup comparisons. This study reported a significant difference in wound depth for the intervention group, but not the control group. The DFU total depth in the intervention group reduced from 0.56 cm (SD, 0.85) to 0.28 cm (SD, 0.38) (*p* = 0.01) compared with the control group, which increased from 0.61 cm (SD, 0.84) to 0.80 cm (SD, 1.26) (*p* = 0.37) [44].

### Adherence to exercise

Two studies measured participants’ self reported adherence to exercise [43, 44]. One study reported 20% of participants exercising two times per day, 20% of participants ceasing exercise after 8 weeks, 20% of participants not reporting frequency, 10% of participants exercising one time per day, 10% of participants exercising two times every third day, 10% not exercising at all and 10% exercising three times per day [43].

A second study reported 26.7% of participants exercising between 0 and 30 days, 50% of participants exercising between 31 and 60 days and 23.3% of participants exercising between 61 and 90 days [44].

A third study did not measure adherence to exercise, as the intervention was conducted in a supervised setting [45].

### Complication and adverse events

One adverse event was recorded across the three studies in which a participant from the intervention group experienced wound deterioration due to osteomyelitis [43].

## Discussion

All three studies included in this review utilised non-weight bearing exercises as the intervention. While the search strategy was designed to capture studies utilising all forms of exercise (i.e. weight bearing and non-weight bearing), this result may be due to weight bearing exercise being considered detrimental to healing of DFUs [1, 17].

This review found a mixture of positive and inconclusive results to support non-weight bearing exercise as an intervention to improve healing of DFUs. One of the included studies [43] was conducted in the form of a pilot study. The small sample sizes of the study may have affected the reliability of the study’s results, and therefore, the results of this study should be interpreted with caution. While a second study [44] achieved statistically significant results with respect to total wound size reduction, these findings should also be interpreted with caution as the results were analysed within each treatment group and not between the treatment groups. A third study reported statistically significant percentage reduction of wound sizes [45]. There were no reported deaths and no reported minor or major amputations as a result of the exercise intervention. There was one adverse event recorded in which a participant in the intervention group experienced wound deterioration due to developing osteomyelitis [43], but this was not deemed related to the prescribed intervention.

The results of this systematic review support supervised exercise programs in preference to unsupervised exercise programs completed in the home setting. While unsupervised exercise programs in the home setting are beneficial in that they are low cost, accessible, safe and easy to implement [46], adherence may potentially be an issue and is influenced by multiple factors such as age, motivation, believing in its benefits, follow ups and the complexity of exercises prescribed [47]. Two studies [43, 44] measured self-reported adherence to non-weight bearing exercise in the home setting in the form of an exercise log, where participants were required to record the type and number of exercises completed. This review found that participants were more likely to experience issues with maintaining accurate records relating to their adherence to exercise when participating in an unsupervised exercise program [43, 44], which is similar to the findings of a study by Anar [47]. A large component of an unsupervised exercise program relies on the participant’s self-motivation, which may be variable [48], their ability to perform the exercise independently as well as accurately reporting exercise frequency [49].

While unsupervised home exercises may be more accessible for patients, this review found that adherence and outcomes were more favourable when exercises were performed in a supervised setting. Exercise conducted in a supervised setting enables a structured program, promotes motivation through visual feedback and encourages participants to achieve the minimum required dose of exercise [50]. However, supervised exercise programs also present with barriers including difficulty in the initial set up of a program, costs, availability of classes and limits on the number of participants allowed to engage per class [51].

Currently, patients presenting with DFUs are discouraged from weight bearing activity in order to minimise plantar pressure to the ulcer site [1, 17]. Of concern is that all types of exercise may then be avoided, despite the fact that exercise has numerous benefits for people with diabetes, including improvement of blood sugar control and lipid profile [52–54]. The findings of this systematic review suggest that non-weight bearing exercises may be safely utilised as part of the management and treatment plan for patients with DFUs and may potentially be beneficial for wound healing [43–45]. Non-weight bearing exercise programs should be designed by a dedicated health professional and DFUs should be closely monitored.

To our knowledge, this is the first systematic review to investigate the effects of exercise and healing of DFUs. The strengths of this review include a rigorous inclusion and exclusion criteria, search strategy, and the included studies were systematically selected, reviewed and assessed by two independent reviewers using standardised methods.

Limitations of this review include the small number of studies, small sample sizes, different participant characteristics at baseline, and moderate quality of studies. Furthermore, the prescribed exercises were dissimilar and the outcomes of interests were measured differently for each study. As a result, the data could not be pooled in a meta-analysis. The search strategy involved three electronic databases and only English text studies were considered, potentially omitting further RCTs that could have been included in the review. Expanding this systematic review to include all study designs, and not just RCTs, may have resulted in a larger number of included trials. However, the overall quality of included studies may have been negatively impacted.

## Conclusion

There is insufficient evidence from this systematic review to conclusively support exercise as an intervention to improve healing of DFUs. Regardless, the results demonstrate some degree of wound size reduction and there were no negative consequences of the intervention for the participants. Given the potential benefits of exercise on patient health and wellbeing, non-weight bearing exercise should be encouraged as part of the management plan for treatment of DFUs. Further research is required to better understand the relationship between exercise and healing of DFUs. Further high quality RCTs with larger sample sizes and conducted in a supervised environment are required in order to determine the effects of exercise on healing of DFUs. Additionally, the exercise protocol (including the type and frequency) that should be prescribed for these patients remains unclear and requires further investigation.

## Supplementary Information

**Additional file 1: Appendix 1**

**Additional file 2: Appendix 2**

**Additional file 3: Appendix 3**

## Data Availability

The dataset supporting the conclusions of this article is included within the article (and its additional file).

## Abbreviations

DFU: Diabetic foot ulcer
PEDro: Physiotherapy evidence database
RCT: Randomised controlled trial
SD: Standard deviation
