# Supplementary material for: Does exercise improve healing of diabetic foot ulcers? A systematic review
Source: J Foot Ankle Res. 2021 Mar 20;14:19. doi: 10.1186/s13047-021-00456-w (PMC7980337; doi:10.1186/s13047-021-00456-w)
Supplement: Supplementary file 1 — Additional file 1: Appendix 1 [file 13047_2021_456_MOESM1_ESM.docx]

Appendix 1: Medline search strategy

| No. | Searches | Results |
| --- | --- | --- |
| 1 | Diabetes Mellitus/ or Diabetes Mellitus, Type 2/ or Diabetes Mellitus, Experimental/or Diabetes Mellitus, Type1/ | 326196 |
| 2 | Foot/ or Foot Diseases/ or Diabetic Foot/ or Diabetes Mellitus/ or Diabetes Complications/ or Diabetic Neuropathies/ | 193613 |
| 3 | diabet*.ab. or diabet*.ti. | 588889 |
| 4 | diabetic feet.ab. or diabetic feet.ti. | 195 |
| 5 | diabetic foot.ab. Or diabetic foot.ti. | 7408 |
| 6 | 1 or 2 or 3 or 4 or 5 | 673764 |
| 7 | Exercise Therapy/ or Exercise/ or Exercise Test/ | 184569 |
| 8 | Physical Therapists/ or Physical Therapy Modalities/ or Treatment Outcome/ | 947101 |
| 9 | Exercise Therapy/ | 37858 |
| 10 | Exercise/ | 101127 |
| 11 | Resistance Training/ | 7602 |
| 12 | Physical Fitness/ | 26450 |
| 13 | exercis*ab. or exercis*.ti | 276438 |
| 14 | physical therap*.ab. or physical therap*.ti. or physiotherap*.ab. or physiotherap*.ti. | 43279 |
| 15 | physical activit*.ab. or physical activit*.ti. | 101389 |
| 16 | aerobic exercis*.ab. or aerobic exercis*.ti. | 8715 |
| 17 | resistance exercis*.ab. or resistance exercis*.ti. | 5279 |
| 18 | physical train*.ab. or physical train*.ti. | 5604 |
| 19 | exercise therap*.ab. or exercise therap*.ti. | 3090 |
| 20 | 7 or 8 or 9 or 10 or 11 or 12 or 13 or 13 or 14 or 15 or 16 or 17 or 18 or 19 | 1358866 |
| 21 | Pressure Ulcer/ or Foot Ulcer/ or Ulcer/ or Skin Ulcer/ | 35368 |
| 22 | foot ulcer*.ab. or foot ulcer*.ti. | 5391 |
| 23 | ulcer*.ab. or ulcer*.ti. | 196269 |
| 24 | wound*.ab. or wound*.ti. | 187256 |
| 25 | wound heal*.ab. or wound heal*.ti. | 58175 |
| 26 | foot wound*.ab. or foot wound*.ti. | 168 |
| 27 | 21 or 22 or 23 or 24 or 25 or 25 or 26 | 382777 |
| 28 | 6 and 20 and 27 | 2965 |
